# Body-weight variation as a potential indirect indicator of abdominal fat weight with relevance to laying rate in 90-week-old laying hens

**DOI:** 10.1016/j.psj.2026.107694

**Published:** 2026-08-23

**Authors:** Alexandre Hubert, Nicolas Bedere, Fabien Degalez, Coralie Allain, Lorry Becot, Quentin Berger, Thierry Burlot, Mathieu Emily, Alexandre Ferry, Laetitia Lagoutte, Bénédicte Lebez, Marie-Pierre Sanchez, Thierry Tribout, Frédérique Pitel, Tatiana Zerjal, Sophie Allais, Sandrine Lagarrigue

**Affiliations:** a PEGASE, INRAE, Institut Agro, 35590, Saint Gilles, France; b NOVOGEN, 5 rue des Compagnons, 22960, Plédran, France; c GABI, Université Paris-Saclay, INRAE, AgroParisTech, 78350, Jouy-en-Josas, France; d L'Institut Agro, Université de Rennes, CNRS, IRMAR (Institut de Recherche Mathématique de Rennes)-UMR, 6625, Rennes, France; e GenPhySE, Université de Toulouse, INRAE, ENVT, 31326, Castanet-Tolosan, France

**Keywords:** Laying hen, Body weight variation, Abdominal fat, Genetic association, Advanced age

## Abstract

In livestock species, efficient regulation of body lipid reserves contributes to prolonged reproductive performance, as shown in dairy cattle and more recently in laying hens through abdominal fat reserves. Controlling abdominal fat weight (**AFW**) at 90 weeks may improve metabolic efficiency and late egg production. However, AFW measurement requires slaughter and dissection, preventing routine use. This study therefore evaluated the genetic relationship between adult body-weight variation (**∆BW**) and AFW, and the potential of ∆BW as a non-invasive indirect indicator of AFW in aging hens.

Genetic parameters were estimated for ∆BW between 30 and 70 weeks (**∆BW3070**), 30 and 90 weeks (**∆BW3090**), and 70 and 90 weeks (**∆BW7090**). Strong genetic correlations were observed between AFW and both ∆BW3070 (0.76) and ∆BW3090 (0.78), whereas the estimate for ∆BW7090 was lower and less precise (0.60). ∆BW3090 was positively genetically correlated with laying rate at 90 weeks of age (**LR**, 0.24), similarly to AFW, whereas ∆BW3070 was not (0.03). ∆BW7090 showed a higher positive correlation with LR (0.57), suggesting that late ∆BW may contribute more to the genetic relationship with laying persistence. These results support the evaluation of ∆BW3070 and ∆BW3090 as complementary non-invasive indicators of abdominal adiposity. ∆BW3070 can be obtained 20 weeks before direct AFW measurement, whereas ∆BW3090 shows a stronger genetic relationship with late LR. Their predictive ability and usefulness for selection nevertheless remain to be validated.

Genome-wide association studies identified three QTLs for ∆BW3070 and two for ∆BW3090, none of which overlapped with the AFW QTLs previously detected in this population. LD-guided refinement reduced the size of some QTL regions by more than 40 %, narrowing one region to only four candidate genes. An eQTL approach also highlighted four additional candidate genes likely regulated by common variants, but these associations and colocalizations do not establish causal effects.

Overall, ∆BW3070 and ∆BW3090 are potential non-invasive indicators of AFW in this population. However, external validation and evaluation of predictive ability and correlated selection responses are required before implementation.

## Introduction

In laying hens, identifying the genetic mechanisms that influence egg production during the late laying period could help improve long-term productivity. Among the key biological processes that may support persistence, lipid metabolism has been proposed as a key player. In livestock species, efficient regulation of body lipid reserves has been shown to support prolonged reproductive performance, as demonstrated in dairy cattle (Friggens et al., 2013; Vallimont et al., 2011), and more recently, in laying hens by examining abdominal fat reserves (Hubert et al., 2026). Indeed, egg production demands energy (Barzegar et al., 2019), particularly for yolk formation, which contains approximately 30 % lipids (Ahn et al., 1997). Although body lipid reserves provide an important energy source for egg production, excessive fat accumulation may impair laying performance and reproductive efficiency (Kochetova et al., 2026). Direct measurement of abdominal fat weight (AFW) requires slaughter and dissection and cannot be applied routinely. The present study therefore evaluated whether body-weight variation could provide a potential non-invasive indirect indicator of AFW. Because AFW was previously genetically correlated with late laying rate, relationships between body-weight variation and laying rate were also examined to assess the relevance of this potential indicator to prolonged reproductive performance.

Body weight is a composite trait reflecting the combined contributions of fat mass, lean tissues, organs, the reproductive tract, skeletal tissues, digestive contents, and hydration. In broiler breeder hens, adult body-weight gain can be accompanied by increased fat deposition whereas lean mass remains comparatively stable, but both components vary during production (Salas et al., 2019). Comparable longitudinal body-composition changes remain limited in adult laying hens. Nevertheless, sexual maturity is known to induce major metabolic changes, notably estrogen-stimulated hepatic lipogenesis and increased lipid deposition to support egg production (Hermier, 1997; Leclercq, 1984). This provides a biological rationale for investigating whether changes in fat deposition contribute sufficiently to body-weight variation (∆BW) for the latter to serve as a non-invasive indirect indicator of abdominal fat weight. To test this hypothesis, genetic parameters were estimated between fat reserve traits and ∆BW. Fat reserves were assessed using two traits recorded at 90 weeks: AFW and back fat thickness (**BFT**), the latter being recognized as a reliable proxy for overall adiposity (Bédère et al., 2024). Three **∆**BW traits were examined: body weight variation between 30 and 70 weeks (**∆BW3070**), corresponding to the transition from early to mid-lay, body weight variation between 30 and 90 weeks (**∆BW3090**), capturing changes over an extended production period, and body weight variation between 70 and 90 weeks (**∆BW7090**), reflecting body weight changes occurring specifically during the late laying period. If these temporal body weight change measures reflect true changes in fatness, they could offer simple, non-invasive indirect indicators of adiposity suitable for large-scale phenotyping.

Genome-wide association studies (**GWAS**) for **∆BW** traits were performed, as previously done in the same population for AFW (Hubert et al., 2026) and BFT (Bédère et al., 2026) to identify potential common **QTL** (Quantitative Trait Loci) between fat reserve traits and ∆BW. GWAS scan the genome at high resolution to detect variants statistically associated with phenotypic variation but do not identify causal variants by themselves. GWAS studies have identified thousands of genomic loci associated with a wide range of complex traits and diseases (Alonso et al., 2021). Most associated variants are located in non-coding regions and are thought to influence gene regulation rather than protein structure (Hindorff et al., 2009; Manolio et al., 2009). This regulatory nature of loci detected by GWAS makes the identification of causal variants particularly challenging, as our knowledge of regulatory variants across the genome remains limited. GWAS were integrated with expression quantitative trait loci (**eQTL**) analyses, which identify regions associated with variation in gene expression. Colocalization of QTL and eQTL signals can support, under its modeling assumptions, the hypothesis that two association signals share a causal variant, but it does not establish causality. However, because gene expression is highly context-dependent, the choice of tissue and age is critical. Liver was selected because of its central role in lipid synthesis and export in laying hens. Nevertheless, adipose tissue is also biologically relevant as the principal storage compartment and a site of adipogenic and lipolytic regulation (Wang et al., 2017). Thus, integrating QTL and hepatic eQTL signals may help prioritize candidate genes under the hypothesis that regulatory variants contribute to interindividual variation in both hepatic gene expression and adiposity at advanced ages. However, this liver-based approach does not capture regulatory variants acting specifically in adipose tissue or other organs.

## Materials and methods

### Animals

A total of 9,657 laying hens were considered in our study. All hens originated from a pure commercial Rhode Island Red line provided by the breeding company Novogen (Plédran, France) and were distributed across 17 batches (named A to Q), hatched and reared at different times (around 2 batches per year). The rearing conditions were the same as those described in Hubert et al. (2026). Until 65 weeks of age, all hens from six batches (H, J, L, N, P and Q) were reared on the floor, whereas animals in other batches were reared either on the floor or in collective cages. At 65 weeks of age, all hens were transferred to individual cages, allowing the recording of individual performances up to 90 weeks of age. Breeding conditions included a lighting regime of 16 h of light per day and ad libitum access to a commercial feed (11.47 MJ/kg of metabolizable energy, 16.5 % of crude protein, and 3.70 % of calcium). All hens were genetically related and originated from a hierarchical mating design, with each sire mated to an average of four dams. Depending on the phenotype considered, the number of dams with phenotyped offspring per trait ranged from 380 to 1,818 and the number of sires with phenotyped offspring ranged from 168 to 625. In the statistical models, batch was included as a factor to account jointly for differences in season, management conditions during the period, and generation. Hatch date was not retained because its effect was not statistically significant. Because season, management period, generation, and potentially part of the temporal genetic trend were confounded within batches, their respective contributions could not be estimated separately.

### Phenotypes at 90 weeks of age

Abdominal Fat Weight (AFW) was measured on the same subset as in Hubert et al. (2026). Hens were slaughtered by neck cutting and bleeding, immediately after electrical stunning. AFW was then measured on 1,113 hens from four batches (I, M, N and Q). Back fat at 90 weeks of age (**BFT**) was measured by ultrasound in 1,513 hens, in three batches (M, N and O), as described in Bédère et al. (2024). In addition, the difference in body weight between 30 and 70 weeks of age (∆BW3070) was measured on 9,657 hens in the 17 batches (A-Q) and the differences in body weight between 30 and 90 weeks of age (∆BW3090) and between 70 and 90 weeks of age (∆BW7090) were measured on 1,658 and 1,653 hens in four batches (M, N, O and Q). Laying rate (LR) was measured in 6,733 hens from 12 batches (F to Q). LR was calculated as described in Hubert et al. (2026), by dividing the number of eggs laid from 85 to 95 weeks of age by the number of days in this period, with a minimum duration of two weeks of measurement. Hens with an LR below 50 % or experiencing a laying pause, defined as no egg laid for seven consecutive days, were excluded to restrict the analysis to hens that remained actively laying. This criterion was intended to distinguish quantitative variation among active layers from laying cessation potentially associated with heterogeneous and unrecorded biological states, such as temporary reproductive interruption, molting, stress, or health and reproductive disorders. Consequently, the estimated genetic correlations are conditional on active laying status and should not be extrapolated to the entire population of aged hens.

### Transcriptome at 90 weeks of age

Hepatic transcriptomes from 459 laying hens belonging to three batches (I, M and N), as described in Hubert et al. (2026), were sequenced. Illumina RNA sequencing (**RNA-seq**) was performed using the TRUSEQ stranded total RNA procedure generating around 100 million paired-end 150 bp reads per sample. Sequencing data (FASTQ files) were mapped to the GRCg7b reference genome (GCF_016699485.2). Gene expression was quantified using an enriched gene annotation (Degalez et al., 2024). This annotation contains 24,102 protein-coding genes (**PCG**s) and 44,428 long non-coding RNAs (**lncRNA**s). Alignment and expression quantification were performed using the *nf-core* “rnaseq” v3.8.1 pipeline with the *STAR-RSEM* aligner (Ewels et al., 2020), which provided raw read counts and transcripts per million (**TPM**). Raw counts were normalized using the trimmed mean of M-values (**TMM**) scaling factor method (Robinson and Oshlack, 2010) using the *R* package *edgeR* (v3.32.1) (Robinson et al., 2010). Library size normalization was performed using the *calcNormFactors* function and expression values were scaled by gene length with the *rpkm* function. A gene was considered expressed if it had at least six reads and a normalized expression level of ≥ 0.5 TMM in at least 75 % of the samples. These transcriptomic data were used to quantify gene expression levels for downstream integrative analyses, including SNP calling from RNA-seq reads and hepatic eQTL mapping for candidate regulated gene prioritization. Raw RNA-seq data are publicly available through the ENA and FAANG portals under accession numbers PRJEB63475.

### Genotypes using the chicken 10k, 60k & 600k arrays

Animals were genotyped by Novogen, as described in Hubert et al. (2026), using three SNP chips of different marker densities according to their generation. The oldest animals (batches A to I) were genotyped using the 600k AFFYMETRIX AXIOM High-Density (**HD**) array (Kranis et al., 2013), whereas the subsequent generations (batches J to P) were genotyped using a 60k medium-density (**MD**) SNP chip (Novogen proprietary chip; Herry et al., 2018). The youngest animals (batch Q) were genotyped using a 10k low-density (**LD**) SNP chip (Novogen proprietary chip). These three arrays were originally designed on the Galgal5 assembly and SNP coordinates were subsequently converted to the last up-to-date GRCg7b assembly using the NCBI *Remap* and UCSC *Liftover* tools. Before imputation, a quality control was performed on autosomal SNPs using *PLINK* v1.9 (Chang et al., 2015). Four successive quality control filters were applied: i) SNP call rate ≥ 95 %, ii) individual call rate ≥ 95 %, iii) minor allele frequency (**MAF**) ≥ 5 %, and iv) Hardy-Weinberg equilibrium probability ≥ 10^−4^. A stepwise imputation strategy was adopted to account for both population structure across generations and differences in marker density. It was conducted in three successive steps as described in Hubert et al. (2026) using *FImpute2* (Sargolzaei et al., 2014) and restricted to autosomal chromosomes. Parentage errors with a rate ≥ 0.05 were identified (param: parentage_test /find_match_cnflt /ert_mm=0.05) and random allele filling was disabled. The 818 individuals from the last batch (batch Q), genotyped using the LD SNP chip, were imputed to MD using a reference population of 6,931 individuals from batches M to P genotyped at 60k. Individuals from batches J to L genotyped at 60k were imputed to HD using a reference population composed of animals from batches A to I, all genotyped at HD. Finally, the 7,749 individuals obtained from the first MD imputation step (batches M to Q) were imputed to HD using the 7,407 individuals from the second imputation step (batches A to L) as the reference population. Imputation accuracy is known to decrease with increasing generational distance between reference and target individuals, making it essential to minimize this distance in populations with many successive generations (Herry et al., 2018). In addition, progressive increases in marker density improve haplotype reconstruction compared with direct imputation from low- to high-density genotypes (Van Binsbergen et al., 2014). Study-specific masking-based imputation accuracy was not estimated for the present dataset. In an earlier validation of the low-density design in the same Rhode Island Red line, the mean correlation between the imputed 10 K genotypes and the HD genotypes was greater than 0.95 (Herry et al., 2018). These published values support the selected strategy but are not accuracy estimates for the present imputed dataset. In the end, 287,607 genotypes were obtained for 15,317 individuals (9,532 males and 5,785 females) and used for further analyses.

### SNP-calling from RNA-seq data

As described in Hubert et al. (2026), SNP calling from RNA-seq data was performed using the *nf-core “rnavar”* pipeline (version 1.0.0) (Ewels et al., 2020). A population-level .vcf file was obtained by combining per sample .gvcf using the *CombineGVCFs* function from *GATK 4.4.0.0* (Van der Auwera and O’Connor, 2020), followed by the *GenotypeGVCFs* function with the option *-stand-call-conf 20*. Variants were then filtered using the *SelectVariants* function, retaining only bi-allelic SNPs with the options *-select-type-to-include SNP* and restrict alleles to *BIALLELIC*. Hard filtering was applied using the *VariantFiltration* function adapting the *GATK* recommendations (Fisher Strand bias > 30 and Quality by Depth < 2). These filtering steps resulted in a total of 10,994,047 SNPs. Only SNPs genotyped in at least 80 % of individuals, with a sequencing depth ≥ 10 reads and a MAF ≥ 0.05, were retained regardless of the associated genotype. This final filtering yielded 106,962 SNPs that were included in downstream analyses.

### Heritabilities and genetic correlations

Genetic parameters of the six traits (∆BW3070, ∆BW3090, ∆BW7090, AFW, BFT and LR) were estimated using average information restricted maximum likelihood (**AIREML**) with two multi-trait animal linear mixed models, based on all available information from the 17 batches. Because ∆BW3070, ∆BW3090, and ∆BW7090 were highly genetically correlated, fitting the three traits simultaneously resulted in convergence issues. Therefore, the analyses were performed using two separate models: the first including ∆BW3070, ∆BW3090, AFW, BFT and LR, and the second including ∆BW3070, ∆BW7090, AFW, BFT and LR. The environmental effects included in the fixed part of the mixed models were first identified for each trait using a simplified mixed-effects model fitted with the *nlme* procedure in *R* (Pinheiro et al., 2025) including the random effect of animals sire. The environmental effects tested were the combined effect of batch and rearing system before 65 weeks of age, the location of the cage within the building, and body weight at 30 weeks of age (**BW30**) (tested only for AFW and BFT to account for differences in body size). The location of the cage within the building refers to the battery cage system, divided into two sides, itself divided into eight groups of cages, accounting for potential micro-environmental variation within the building. BW30 was used as a covariate to account for structural body size prior to the onset of late laying. Using body weight at slaughter could introduce bias, as it is itself influenced by fat deposition and metabolic status at the time of measurement, potentially leading to over-adjustment. BW30 therefore provides a proxy for baseline body size independent of later metabolic changes. In this preliminary model, sire was included as a random effect to account for the main population structure. The combined effect of batch and rearing system before 65 weeks of age and the effect of cage location were significant (p-value ≤ 0.05) for ∆BW3070, ∆BW3090, ∆BW7090, AFW, BFT and LR, whereas BW30 was significant for AFW and BFT. Fixed effects found to be significant in this simplified model were subsequently retained in the variance component estimation model described below. Outliers, defined as observations whose residuals from this simplified model exceeded ± 4 standard deviations, were not considered in subsequent analyses. The multi-trait models considered for variance and covariance component estimation were the following:

Y=Xβ+Zu+e

where Y is the vector of phenotypic records for the traits; β is the vector of fixed effect coefficients associated with the significant cofactors and covariates identified for each trait; $u\;∼N(0,\;VC_{g}⊗A)$ is the vector of random additive (polygenic) effects for the considered traits with A the pedigree relationship matrix, *VC_g_* the matrix containing the traits additive genetic variances and covariances, and ⊗ represents the Kronecker product; and $e\;∼N(0,\;VC_{e}⊗I)$ is the vector of residual effects with I the identity matrix and *VC_e_* the matrix containing the traits residual variances and covariances; X and Z are corresponding incidence matrices, in particular Z is the incidence matrix relating observations to breeding values. The pedigree was traced back over 19 generations, resulting in a total of 81,836 individuals included in the pedigree file. The software AIREMLF90 (Misztal et al., 2022) was used to estimate heritabilities and genetic correlations between traits.

### Genome-wide association study (GWAS)

The 287,607 imputed genotypes were tested for association with ∆BW3070, ∆BW3090 and ∆BW7090 in separate analyses using the *GCTA* software (Yang et al., 2011). The following linear mixed model accounting for population structure through a genomic relationship matrix was applied:

$$Y^{*}=\;\mu +X\beta +\mathrm{Zu}\;+\;e$$

Where $Y^{*}$ is the vector of phenotypes pre-adjusted for solutions for fixed effects estimated in the simplified mixed model including random sire effect; μ is the population mean of pre-adjusted phenotypes; X is the vector of imputed genotypes for the tested variant, coded as 0, 1 or 2; β is the allelic substitution effect of the tested variant; Z is the incidence matrix relating observations to breeding values (random animal effects) (here ***Z*** = ***I***_n_, the identity matrix of dimension n×n, where n is the number of individuals, as each animal contributed a single record); $u∼N(0,\;G\;\sigma_{u}^{2})$ is the vector of random polygenic effects, with G the genomic relationship matrix based on 287,607 autosomal SNPs with MAF > 0.05 and $\sigma_{u}^{2}$ the polygenic variance; and finally, e is the vector of residual effects. Principal components were not included as fixed covariates because the linear mixed model based on a genomic relationship matrix implicitly accounts for both family relatedness and population structure in a genetically homogeneous purebred population (Yang et al., 2011). The adequacy of this correction was empirically verified for each trait through QQ plots and genomic inflation factors (λ ranging from 0.98 to 1.03), which did not indicate substantial inflation.

As described in Hubert et al. (2026), the GWAS significance threshold was determined by identifying independent SNPs (Jiang et al., 2025) using the *PLINK* v1.9 *–indep-pairwise* function, with a window size of 1 Mb, corresponding to the QTL region size, a step size of 5 SNPs and a linkage disequilibrium (**LD**) threshold of 0.3, as previously demonstrated in chicken (Fu et al., 2015) and also applied in human studies (Ardlie et al., 2002). This approach was used to estimate the effective number of independent tests and to avoid an overly conservative multiple testing correction due to LD between markers. This LD-adjusted Bonferroni approach yields a less conservative threshold than applying Bonferroni correction to all 287,607 tested SNPs, and still controlling the family-wise error rate based on the effective number of independent tests (Li and Ji, 2005). This strategy has been applied in multiple GWAS studies of which some in chickens using the same 600 K SNP array (Hubert et al., 2026; Liu et al., 2019; Marchesi et al., 2021). Of the 287,607 SNPs from the 600k array in the population, 3,533 SNPs were found to be independent. The genome-wide significance threshold was therefore set at $1.42\;\times \;10^{-5}$, corresponding to a multiple testing Bonferroni correction, with a 5 % error rate (0.05/3,533). GWAS signals were considered suggestive when their p-values were lower than $5.66\;\times \;10^{-5}$ (0.2/3,533), corresponding to a 20 % error rate. As a sensitivity analysis, results were also compared with the conventional Bonferroni threshold based on all 287,607 markers (0.05/287,607 = 1.74 × 10⁻⁷). QTLs for which no continuum of decreasing p-values was observed around the lead SNP were not considered, as this criterion was used to distinguish true association signals supported by local LD structure from isolated non-supported associations. For each trait, Manhattan and quantile-quantile (**QQ**) plots were generated using the *R* package *qqman* (Turner, 2018) to visualize the distribution of association signals on the genome and to evaluate potential inflation due to population structure or other confounding factors. The genome-wide inflation factor **λ** was calculated as the ratio of the median observed **χ²** statistic to 0.456, which corresponds to the expected median **χ²** value under the null hypothesis of no inflation (Devlin and Roeder, 1999).

### eQTL detection

As described in Hubert et al. (2026), associations between SNP genotypes and gene expression data were estimated with the *TensorQTL* software (Hore et al., 2016). Two categories of regulatory associations were analyzed. Local (cis) eQTLs were defined as associations between a gene’s expression and SNPs located within ±500 kb of its transcription start site (**TSS**). All remaining SNP-gene pairs, including those located on different chromosomes, were considered distant (trans) eQTLs. To ensure robust detection of eQTL signals and to limit the inclusion of isolated lead SNP associations, only eQTLs for which at least five SNPs within a 100 kb window exceeded the significance threshold and were in linkage disequilibrium with the lead SNP (LD ≥ 0.5) were retained. eQTL mapping was performed with inverse-normal transformed gene expression values (GTEx Consortium et al., 2020; Taylor-Weiner et al., 2019) and a combined genotype dataset integrating SNPs from the 600k commercial array together with additional variants obtained from RNA-seq–based SNP calling, for a total of 382,242 SNPs. The first five principal components (**PCs**) derived from the genotype matrix were included as covariates in the model to correct for relatedness and population structure, and the first five PCs derived from the expression matrix were included to account for environmental and technical variation, following the approach used in human GTEx analyses (GTEx Consortium et al., 2020). These ten covariates were included in all association models. For local eQTLs, the cis module of *TensorQTL* was used to generate gene-level nominal p-values. For distant eQTLs, the trans module was applied to scan all SNP-gene combinations outside the cis window. Significance thresholds were determined according to the number of independent tests performed. For local eQTLs, gene-level significance was assessed using a Bonferroni correction based on the 11,830 expressed genes tested (0.05/11,830). For distant eQTLs, a Bonferroni threshold was applied based on 4,596 independent SNPs (derived from LD pruning) multiplied by the 11,830 expressed genes, corresponding to 54,370,680 independent SNP-gene tests (0.05/54,370,680).

### QTL and eQTL refining

A QTL was initially defined as a 1 Mb region, i.e., ±500 kb around the lead SNP. As described in Hubert et al. (2026), to reduce the size of QTLs, LD among the 287,607 imputed 600k-array SNPs was calculated chromosome by chromosome, using the *–r2* function from *PLINK* v1.9, with parameters *–ld-window-kb 1000000* and *–ld-window 40000* allowing the calculation of LD for all SNP pairs in each chromosome. Refined QTL boundaries were then defined by the most distal SNPs in LD with the lead SNP on both sides, using 0.5 as an LD threshold. The same method was used to refine eQTLs but using LD among the combined set of 382,242 SNPs, comprising imputed 600k-array SNPs and SNPs called from RNA-seq data.

### QTL and eQTL colocalization

As described in Hubert et al. (2026), colocalization between refined QTLs and eQTLs was considered only when the loci physically overlapped, ensuring that both signals mapped to the same genomic interval. Colocalization was then evaluated based on the relationship between the QTL and eQTL lead SNPs. These two SNPs had to be identical or having a strong pairwise LD of at least 0.7, meaning they belonged to the same LD block. To visualize the LD blocks, LD structure was illustrated by a color scale representing the LD between each SNP and the corresponding lead SNP. Colocalization was then supported by the presence of SNPs displaying similar LD values, reflected by comparable colors, at matching genomic positions across QTL and eQTL regions. Similar LD patterns at matching genomic positions were treated as descriptive support for compatible association signals, not as evidence of causality.

To complement the LD-pattern comparison, colocalization between each QTL and eQTL was also assessed using the Bayesian framework implemented in the *R* package *coloc* (Giambartolomei et al., 2014). This approach evaluates whether two association signals detected in the same genomic region are statistically consistent with a shared underlying causal variant, based on summary statistics and the model assumptions. For each tested gene, analyses used SNPs common to the QTL and eQTL datasets within the local regulatory region. Effect estimates, standard errors, allele frequencies, and sample sizes were provided for both quantitative traits. The *coloc* method evaluates five hypotheses: (H0) neither the QTL nor the eQTL is associated with any variant in the region; (H1) only the QTL is associated; (H2) only the eQTL is associated; and (H3) both traits are associated but are driven by distinct causal variants. In addition, a fifth hypothesis (H4) represents the scenario in which both traits are associated and share a single common causal variant. Posterior probabilities (**PP**) were computed for each hypothesis. An H4 posterior probability greater than 0.8 was considered strong statistical support for colocalization, rather than proof of causality (Boltz et al., 2024; Gudjonsson et al., 2022; Wu et al., 2019).

### Functional candidate gene exploration

As described in Hubert et al. (2026), to identify functional candidate genes, genes associated with lipid metabolism within each QTL were identified through a keyword-based search applied with the user-friendly GEGA tool (https://gega.sigenae.org/) and linked to the enriched gene atlas (Degalez et al., 2024). Functional annotations in GEGA are primarily derived from Gene Ontology (GO) terms, one of the most comprehensive and widely used resources for functional gene annotation (Ashburner et al., 2000; The Gene Ontology Consortium et al., 2026). The keywords used were: "lip", "chol", "fat", "steato", "nash", "nafld", and "adip". A total of 4,220 genes were labelled as associated with lipid metabolism based on the presence of these keywords in their annotation.

## Results

### Distribution and descriptive statistics of phenotypes at 90 weeks of age

Distribution and descriptive statistics were computed for each trait to assess the range and phenotypic variability at 90 weeks of age (Fig. 1).

**Fig. 1:**
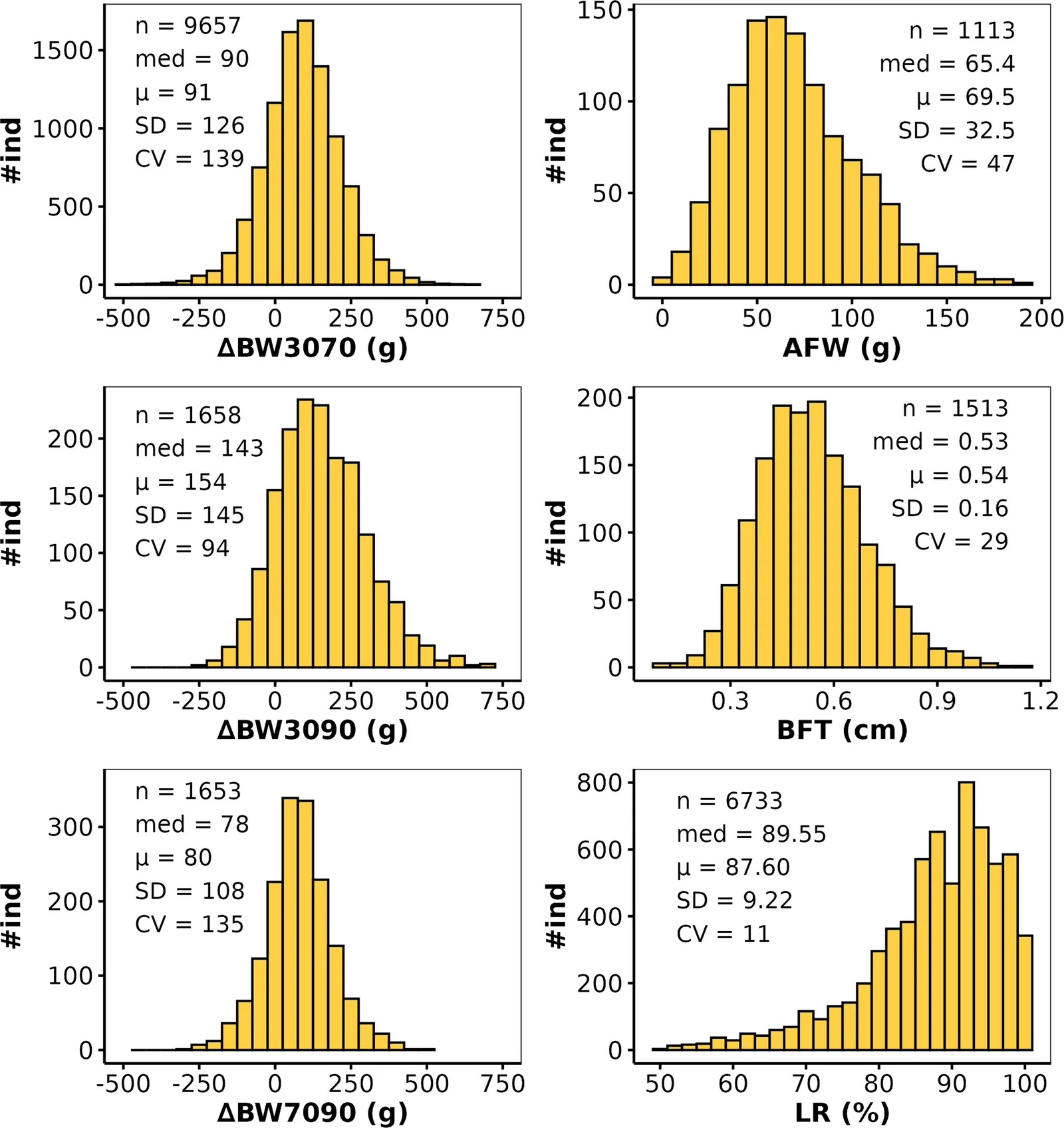
Phenotypic distributions of the six traits. ∆BW3070 stands for body weight variation between 30 and 70 weeks of age; ∆BW3090 stands for body weight variation between 30 and 90 weeks of age; ∆BW7090 stands for body weight variation between 70 and 90 weeks of age; AFW stands for abdominal fat weight at 90 weeks of age; BFT stands for back fat thickness at 90 weeks of age; LR stands for laying rate at 90 weeks of age; n stands for the number of records; med stands for median; µ stands for mean; SD stands for standard deviation; CV stands for coefficient of variation (%); #ind stands for number of individuals.

### Genetic parameters

Heritabilities, genetic correlations and phenotypic correlations for ∆BW3070, ∆BW3090, AFW, BFT and LR are reported in Fig. 2A. AFW and BFT showed high heritabilities (0.46 and 0.47), whereas ∆BW3070 and ∆BW3090 exhibited lower heritabilities (0.26 and 0.37). Both the phenotypic and genetic correlations between ∆BW3070 and AFW (0.64 and 0.76) and ∆BW3090 and AFW (0.73 and 0.78) were strong and positive, supporting the evaluation of the two ∆BW traits as genetically correlated indicators of AFW in this population. Genetic correlations between these two ∆BW traits and BFT were lower (0.45 and 0.47). AFW was positively genetically correlated with BFT (0.67) and LR (0.26). ∆BW3090 was also positively genetically correlated with LR (0.24), whereas ∆BW3070 was not (0.03).

**Fig. 2:**
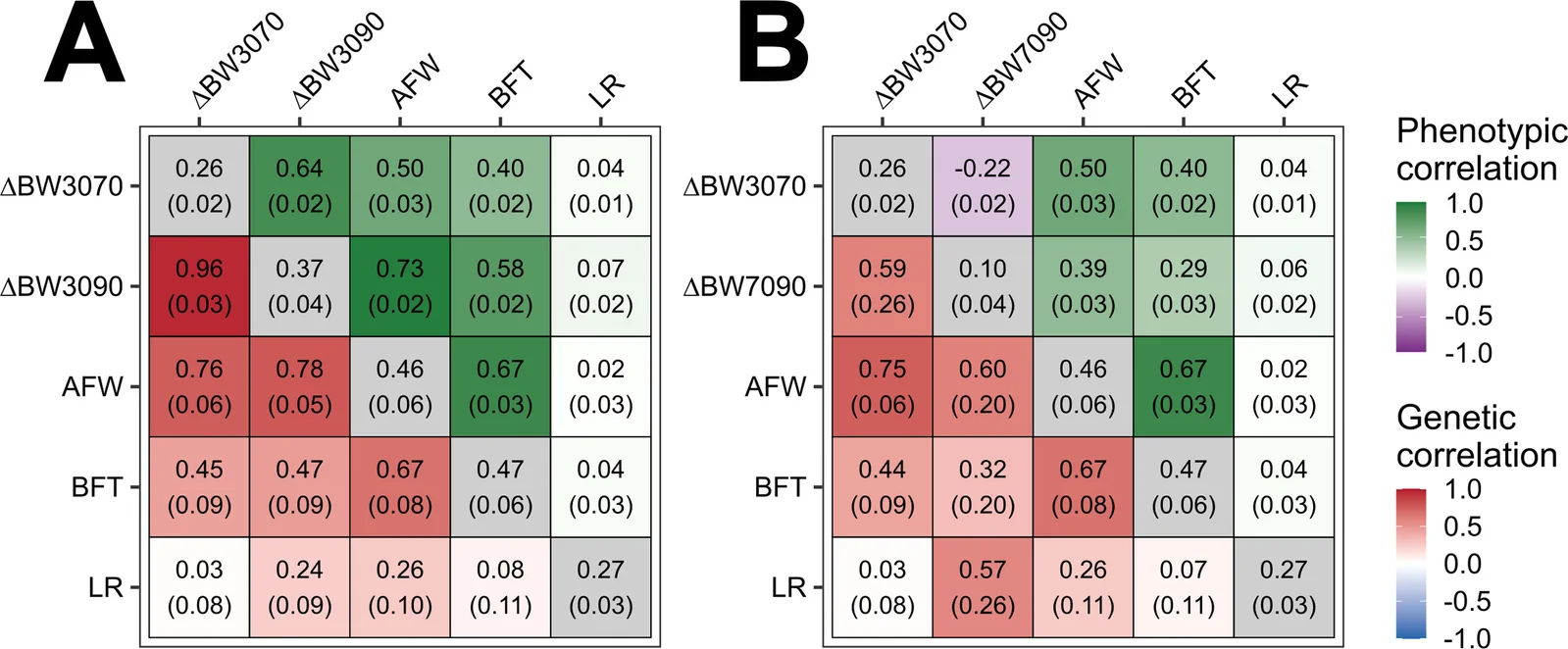
Estimates of genetic parameters for different ∆BWs, lipid reserves and the laying rate. A) ∆BWs are ∆BW3070 and ∆BW3090 and B) ∆BWs are ∆BW3070 and ∆BW7090. Heritability on the gray diagonal, genetic correlations below the diagonal and phenotypic correlations above the diagonal. The standard errors of the estimates are indicated in parentheses. ∆BW3070 stands for body weight variation between 30 and 70 weeks of age; ∆BW3090 stands for body weight variation between 30 and 90 weeks of age; ∆BW7090 stands for body weight variation between 70 and 90 weeks of age; AFW stands for abdominal fat weight measured at 90 weeks of age; BFT stands for back fat thickness measured at 90 weeks of age and LR stands for laying rate measured at 90 weeks of age.

New estimates of heritabilities, genetic and phenotypic correlations were obtained for the five traits, replacing ∆BW3090 by ∆BW7090 (∆BW3070, ∆BW7090, AFW, BFT and LR), as reported in Fig. 2B. Genetic parameters for ∆BW3070, AFW, BFT and LR were largely unchanged. The heritability of ∆BW7090 was low (0.10). ∆BW7090 is less genetically positively correlated with AFW (0.60) than ∆BW3070. ∆BW7090 is also less genetically positively correlated with BFT (0.32) than ∆BW3070. However, ∆BW7090 is more genetically positively correlated with LR (0.57) than ∆BW3070. In addition, the genetic correlation between ∆BW7090 and ∆BW3070 was positive (0.59), whereas their phenotypic correlation was negative (−0.22). However, the standard errors associated with the ∆BW7090 genetic correlations are relatively high, indicating that these estimates should be interpreted with caution.

### Genome-wide association study (GWAS)

The QQ-plots derived from GWAS results for ∆BW3070, ∆BW3090 and ∆BW7090 indicated no major inflation of test statistics, with genomic inflation factors (λ) equal to 1.01 for ∆BW3070, 1.03 for ∆BW3090 and 0.98 for ∆BW7090, showing that the model fit the data well (Fig. 3). The genomic regions associated with these traits are summarized in Table 1 and the Manhattan plots are provided in Fig. 3. Under the prespecified LD-adjusted threshold, two significant and one suggestive associations were detected for ∆BW3070, on chromosomes 2, 3 and 11, explaining 0.12, 0.15 and 0.12 phenotypic standard deviation, respectively. Two significant associations were detected for ∆BW3090, on chromosomes 1 and 4, explaining 0.22 and 0.20 phenotypic standard deviation. No significant or suggestive QTL was detected for ∆BW7090. No common QTL was detected between ∆BW traits and AFW in this population. Under the conventional Bonferroni threshold based on all 287,607 markers (1.74 × 10⁻⁷), none of the loci reached significance; the lowest observed p-value was 3.09 × 10⁻⁷. The reported loci should therefore be interpreted in relation to the LD-adjusted effective-number-of-tests procedure used as the primary analysis. QTLs for the ∆BW traits were then refined to better prioritize the list of candidate genes.

**Fig. 3:**
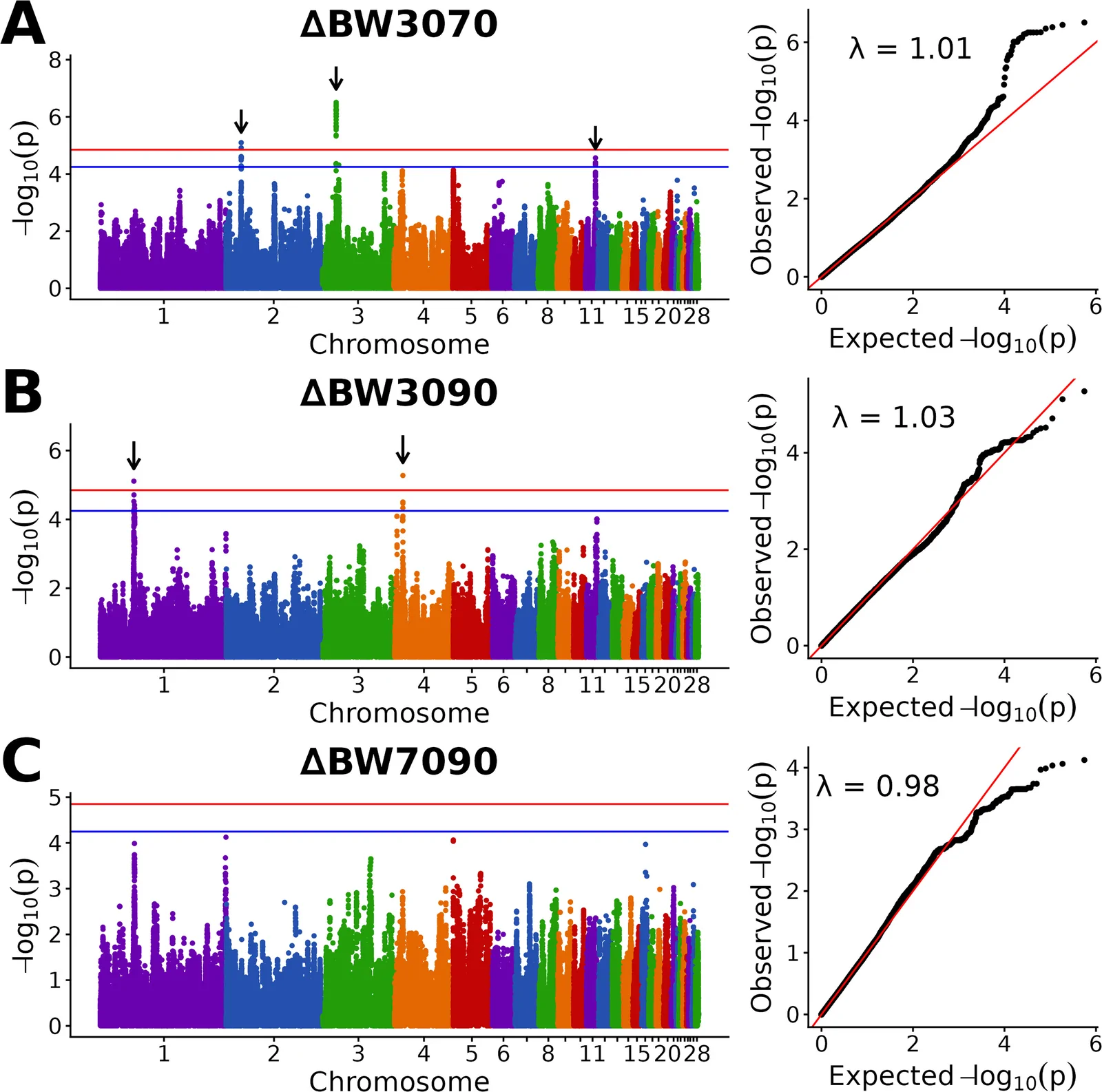
GWAS analysis for ∆BW traits. Manhattan and Quantile-Quantile plots of the GWAS associations for A: Body weight variation between 30 and 70 weeks (∆BW3070); B: Body weight variation between 30 and 90 weeks (∆BW3090) and C: Body weight variation between 70 and 90 weeks (∆BW7090). For all Manhattan plots, the significant threshold was 1.42 × 10^−5^ (red line) and the suggestive threshold was 5.66 × 10^−5^ (blue line). Black arrows indicate the studied QTLs.

**Table 1:** QTLs of abdominal fat weight and body weight variations.

Traits^1^	Chr^2^	Lead SNP (bp)	P-value	MAF^3^	Effect/SD^4^	#Gn^5^	#PCG^6^	lncRNA^7^
∆BW3070	2	23770063	8.00E-06	0.41	−0.12	47	15	30
∆BW3070	3	21251043	3.09E-07	0.24	0.15	62	21	38
∆BW3070	11	15266060	2.77E-05	0.37	−0.12	80	29	40
∆BW3090	1	52028978	7.75E-06	0.32	0.22	92	26	54
∆BW3090	4	12872375	5.20E-06	0.39	0.20	71	23	42

1 AFW stands for abdominal fat weight; ∆BW3070 stands for body weight variation between 30 and 70 weeks of age and ∆BW3090 stands for body weight variation between 30 and 90 weeks of age.

2 Chr stands for chromosome.

3 MAF stands for minor allele frequency.

4 Allele substitution effect of the lead SNP divided by the phenotypic standard deviation (SD) of the trait.

5 Number of genes (Gn) located within the QTL.

6 Number of PCG for protein coding gene.

7 Number of lncRNA for long non-coding RNA.

### LD-guided QTL refining

An LD-based approach was applied to refine each QTL around its lead SNP. Only regions where LD with the lead SNP remained ≥ 0.5 were retained, resulting in reduced QTL intervals. As illustrated in Fig. 4, the QTLs for ∆BW3070 on chromosomes 2 and 3 were reduced by 44 % and 86 % respectively, decreasing the number of genes from 47 to 28 on chromosome 2 and 62 to 4 on chromosome 3. The QTL on chromosome 11 for ∆BW3070 was only reduced by 5 %, which did not narrow down the number of genes in this region. For ∆BW3090, the QTLs on chromosomes 1 and 4 were reduced by 34 % and 41 % respectively, decreasing the number of genes from 92 to 64 on chromosome 1 and from 71 to 42 on chromosome 4. Table 2 presents the refined QTL regions, including their genomic coordinates, physical sizes, and gene content, with the number of PCGs and lncRNAs overlapping each interval.

**Fig. 4:**
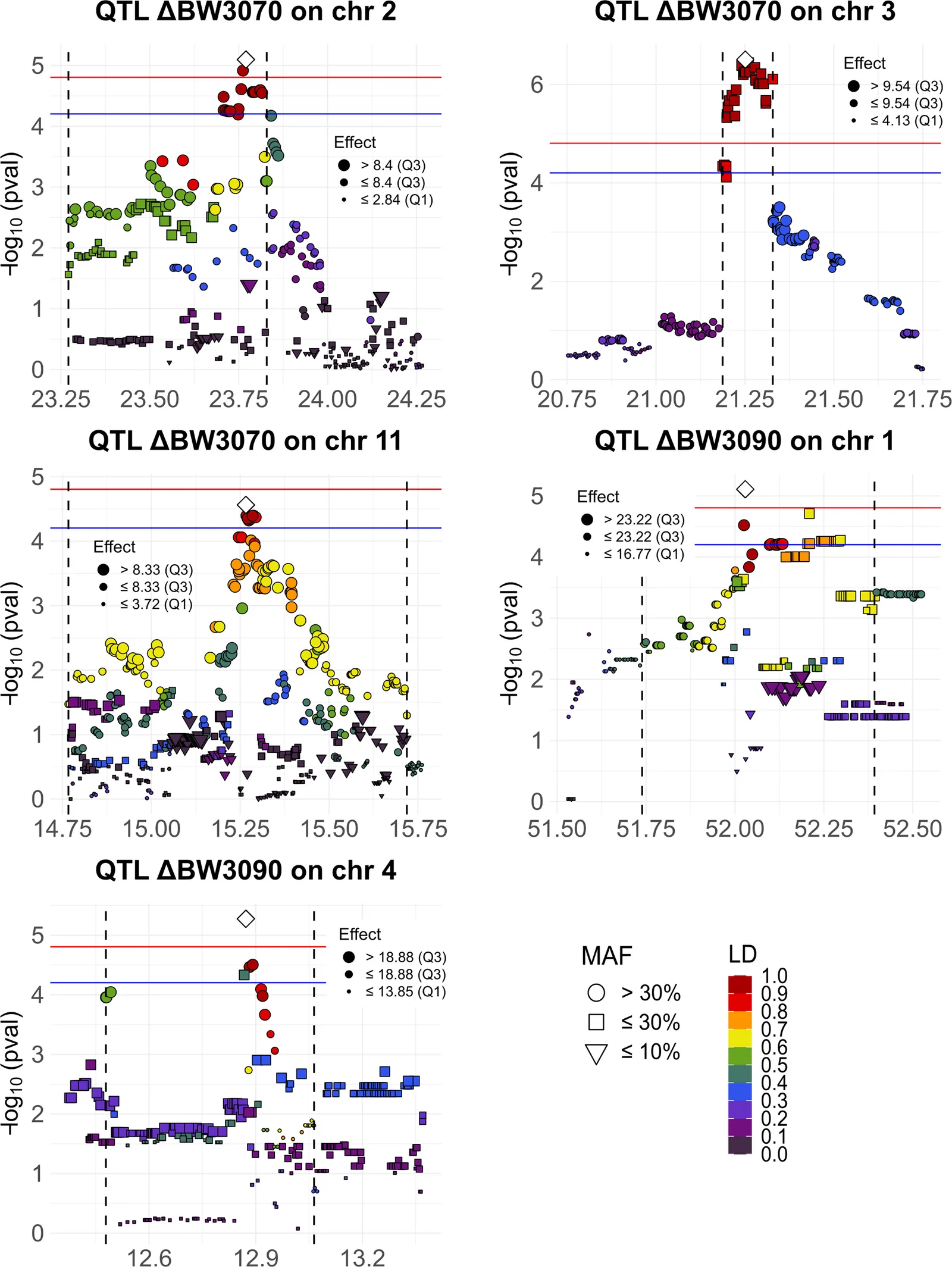
Zoom in ∆BW3070 and ∆BW3090 QTLs. The shape of points corresponds to the Minor Allele Frequency (MAF). Color scale corresponds to the Linkage Disequilibrium (LD) with the leader SNP (r²). The size of points corresponds to the allele substitution effect. The lead SNPs are represented by a white rhombus. The black dashed line represents the reduced interval of each QTL.

**Table 2:** Reduced QTLs of lipid-related traits at 90 weeks.

Traits^1^	Chr^2^	#Gn^3^	%Red^4^	Start (bp)^5^	End (bp)^6^	#Gn: PCG; lncRNA^7^	#GnLip: PCG; lncRNA^8^	#GnLiv: PCG(%); lncRNA^9^	#eGnLiv: PCG(%); lncRNA^10^	#eQTL∼QTL: PCG; lncRNA^11^
∆BW3070	2	47	44	23,271,142	23,828,688	10;16	4 ;0	8 (80);0	6 (75);0	2;0
∆BW3070	3	62	86	21,187,367	21,328,416	0;4	0;0	0;0	0;0	0;0
∆BW3070	11	80	5	14,767,105	15,718,687	29;40	7;0	16 (55);0	11 (69);0	2;0
∆BW3090	1	92	34	51,733,007	52,392,259	16;38	3;0	9 (56);0	3 (33);0	0;0
∆BW3090	4	71	41	12,478,795	13,064,066	13;25	4;0	9 (69);1	3 (33);1	0;0

1 ∆BW3070 stands for body weight variation between 30 and 70 weeks of age and ∆BW3090 stands for body weight variation between 30 and 90 weeks of age.

2 Chr stands for Chromosome.

3 Number of genes within the non-refined QTL.

4 Percentage of the QTL size reduction thanks to linkage disequilibrium refinement.

5 Start of the refined QTL.

6 End of the refined QTL. ^7-11^ The numbers of protein-coding genes (PCGs) and long non-coding RNA genes (lncRNAs) are shown on either side of the semicolon.

7 Number of genes within the refined QTL.

8 Number of genes within the reduced QTL related to lipid metabolism.

9 Number of genes expressed in the liver, the percentage indicates the number of PCGs expressed in the liver relative to the number of PCGs in the region.

10 Number of eGenes (i.e., genes regulated by a local expression QTL (eQTL)) in the liver, the percentage indicates the number of PCG eGenes relative to the number of PCGs expressed in the liver.

11 Number of eQTLs which colocalize with a QTL. QTL.

### Candidate gene prioritization by eQTL integration and functional annotation

Given the strong genetic correlations between ∆BW and AFW and the central role of liver in lipid synthesis and transport, the analysis tested whether variants associated with ∆BW were also associated with hepatic gene expression. For each refined QTL, Table 2 reports the numbers of genes expressed in liver (PCGs and lncRNAs), the numbers of genes regulated in the liver (**eGenes**) with local eQTLs overlapping the QTL, and genes functionally annotated for lipid metabolism. LD-guided refinement and QTL-eQTL colocalization were used to prioritize candidate genes, based on statistical associations and biological support, without demonstrating causality.

Within the refined chromosome 2 QTL for ∆BW3070, four genes had lipid-related functional annotations: *COL1A2, PPP1R9A, PON1*, and *PDK4*. Furthermore, eight genes were expressed in the liver and six of these, *CASD1, PDK4, PON1, BET1, PPP1R9A*, and *LOC420562*, had local eQTLs overlapping the QTL region. Among these six eGenes, *CASD1* and *PDK4* displayed association profiles consistent with colocalization (Fig. 5A) unlike the remaining four eGenes illustrated in Fig. 5B. Their lead eQTL SNPs were in strong LD with the QTL lead SNP (r² = 0.94 for both genes). The coloc analysis supported H4 for *CASD1* (PP.H4 = 0.95) and *PDK4* (PP.H4 = 0.83). Under the single-causal-variant assumption of coloc, these results are statistically consistent with a shared association signal and prioritize *CASD1* and *PDK4* within the region; they do not demonstrate that either gene or variant is causal. Of the two genes, only *PDK4* had a direct lipid-metabolism annotation.

**Fig. 5:**
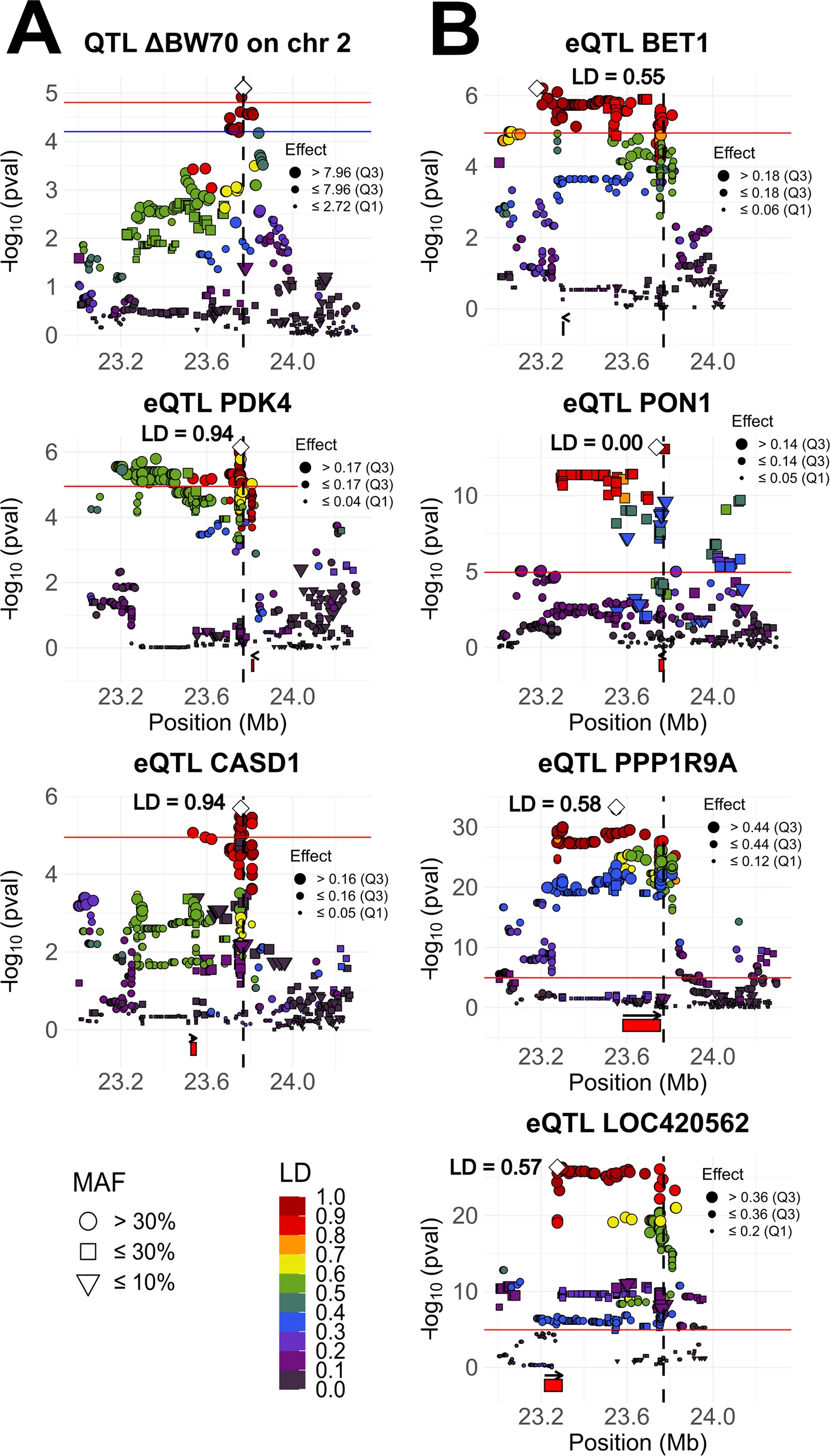
∆BW3070 QTL on chromosome 2 and eQTLs in liver associated with this region. A: ∆BW3070 QTL on chromosome 2 with local eQTLs for *PDK4* and *CASD1*, which co-localized with ∆BW3070 QTL. B: local eQTLs for the eGenes, *BET1, PON1, PPP1R9A* and *LOC420562*, which did not colocalize with the ∆BW3070 QTL. Shape of points corresponds to the Minor Allele Frequency (MAF). Color scale corresponds to the Linkage Disequilibrium (LD) with the leader SNP (r²). The size of points corresponds to the allele substitution effect. The lead SNPs are represented by a white rhombus and their LD with the lead SNP of the ∆BW3070 QTL is indicated next to them. The black dashed line represents the location of ∆BW3070 QTL leader SNP. The red rectangle under each eQTL plot indicates the location of the eGene; the arrow indicates the eGene’s strand.

For the refined chromosome 11 QTL associated with ∆BW3070, seven of the 80 genes had lipid-related functional annotations: *DYNLRB2, ATMIN, GCSH, BCO1, PLCG2, HSD17B2* and *CDH13*. In addition, this refined QTL was found to contain 16 expressed genes in the liver, including six eGenes. Two out of them, *PNAT10* and *CMIP*, displayed GWAS profiles compatible with the QTL signal, and their lead eQTL SNPs were in LD with the QTL lead SNP (r² = 0.73 and 0.67, respectively). The coloc result provided stronger support for H4 for *CMIP* (PP.H4 = 0.88) than for *PNAT10* (PP.H4 = 0.64). Thus, *CMIP* had stronger statistical support, although neither gene has established functional evidence for adiposity regulation in poultry.

The refined chromosome 3 QTL for ∆BW3070 contained four lncRNAs: *FRAGALG000000038861, ENSGALG00010021279, FRAGALG000000014545*, and *FRAGALG000000044730*. None was expressed in liver. According to the GEGA atlas, *FRAGALG000000038861* is expressed predominantly in the ovary.

The refined ∆BW3090 QTLs on chromosomes 1 and 4 contained 64 and 42 genes, of which nine and ten were expressed in liver, respectively. Three and four expressed genes, respectively, were liver eGenes, but none of their eQTLs colocalized with the corresponding QTL. LD between the eQTL and QTL lead SNPs ranged from 0.18 to 0.52. Three genes in the chromosome 1 interval (*MYH9, MB* and *HMOX1*) and four in the chromosome 4 interval (*FGF16, ATRX, ATP7A* and *AMOT*) had lipid-related functional annotations.

## Discussion

### Genetic parameters

The heritabilities of AFW and BFT were relatively high (0.46 and 0.47, respectively) and remained consistent with previous estimates obtained in the same population (Bédère et al., 2026; Hubert et al., 2026), although slight differences were expected because genetic parameters were re-estimated using a different multi-trait model including distinct sets of traits. Heritabilities were lower for ∆BW3070 (0.26) and ∆BW3090 (0.37), but indicate additive genetic variation in both traits. The lower heritability of ∆BW7090 (0.10) indicates less additive genetic variation relative to phenotypic variance. These estimates alone do not quantify realized selection response or breeding value. The heritability reported for body-weight change from 28 to 72 weeks in another laying-hen population was 0.24 (Guo et al., 2023), consistent with the present ∆BW3070 estimate. Comparable estimates for changes between 30 and 90 or 70 and 90 weeks have not been reported.

The pattern of genetic correlations observed in the present study provides important insights into the biological meaning of body-weight variations. AFW was strongly and positively genetically correlated with ∆BW3070 (0.76) and ∆BW3090 (0.78), whereas the estimate for ∆BW7090 was lower (0.60) and less precise. These results suggest that ∆BW3070 and ∆BW3090 may be useful as indirect indicators of AFW in this population. However, predictive ability of these indicators would need independent validation in another population and evaluation of correlated response to selection. AFW was also strongly genetically correlated with BFT (0.67), in agreement with previous findings (Bédère et al., 2026), confirming that both traits share a substantial genetic component of lipid deposition variation. However, BFT exhibited only moderate positive genetic correlations with ∆BW3070 (0.45), ∆BW3090 (0.47) and ∆BW7090 (0.32), markedly lower than those observed with AFW. These results suggest that AFW and BFT represent partially distinct adiposity compartments with different relationships to body-weight variations, with AFW appearing as a more dynamic fat reserve, more responsive to body weight fluctuations than BFT.

Although ∆BW was strongly genetically correlated with AFW, the two traits are not equivalent, as indicated by a genetic correlation below unity. In addition to fat deposition, changes in lean tissues, organs, the reproductive tract, skeletal tissues, digestive contents, and hydration may contribute to ∆BW. Nevertheless, the strong genetic correlations observed between ∆BW and AFW indicate that these traits share a substantial genetic basis, supporting the potential use of ∆BW as an indirect indicator of abdominal fat deposition. Further studies would be needed to determine the respective contribution of non-adipose tissues to the genetic variation in ∆BW.

The association of ∆BW with LR was examined because AFW had previously been positively genetically correlated with LR (0.26) in Hubert et al. (2026), and an indirect indicator considered for breeding should be evaluated in relation to late laying performance. ∆BW3090 showed a positive genetic correlation with LR (0.24), consistent with the correlation previously observed between AFW and LR, whereas the estimate for ∆BW3070 was close to zero (0.03). This pattern suggested that body-weight variation occurring between 70 and 90 weeks of age contributed more strongly to the shared genetic basis with LR than variation occurring earlier in the laying period. Consistent with this hypothesis, the genetic correlation between ∆BW7090 and LR was positive (0.57), although its relatively large standard error (0.26) warrants cautious interpretation. Interestingly, ∆BW3070 and ∆BW7090 showed a positive genetic correlation (0.59), whereas their phenotypic correlation was negative (-0.22). This discrepancy may be explained by the low heritability of ∆BW7090, suggesting a stronger environmental influence on this trait, which can alter the observable relationship between both traits despite a shared genetic basis. The low heritability of ∆BW7090 and the uncertainty surrounding its genetic correlations further limit interpretation of this late body-weight variation.

From a practical perspective, ∆BW3070 can be calculated at 70 weeks, 20 weeks before AFW is directly measured at slaughter, and may therefore provide earlier indirect information genetically related to AFW. In contrast, ∆BW3090 is available only at 90 weeks but also captures body-weight variation during the late laying period and remains genetically correlated with LR. These interval-specific properties could make the two ∆BW traits informative for different breeding objectives, but they do not demonstrate that either trait is an appropriate selection criterion.

Body weights recorded at 30, 70, and 90 weeks could therefore be used to derive ∆BW traits for further evaluation. Where, in most breeding programs, body weight is already routinely recorded at these ages, this could provide indirect information on abdominal adiposity with limited additional phenotyping cost. Before incorporating a ∆BW trait into a global breeding objective, three main aspects should be evaluated. First, its predictive ability for the intended outcome must be established. Second, its genetic correlations with the other traits included in the breeding objective must be characterized to identify potential favorable or antagonistic correlated responses. Third, appropriate weights must be calculated to position the new trait within the global breeding objective and accounting for economic, social, ethical, and environmental considerations. Selection should also target an optimum body-fat level rather than assuming that either continued gain or continued loss is desirable. Potential antagonistic responses and the stability of genetic parameters across lines and environments therefore require evaluation before implementation.

The larger number of ∆BW records and the strong genetic correlations of ∆BW3070 and ∆BW3090 with AFW also support evaluating these traits as potential auxiliary traits for multi-trait genomic prediction of AFW. Correlated auxiliary traits can improve prediction of a less extensively phenotyped target trait under suitable genetic and residual covariance structures (Jia and Jannink, 2012). However, no genomic prediction analysis was performed here. This potential should be tested by cross-validation comparing single-trait prediction of AFW with multi-trait models incorporating ∆BW, followed by validation in an independent population.

### QTL identified for phenotypic traits

As GWAS for AFW and BFT have already been reported (Bédère et al., 2026; Hubert et al., 2026), the present GWAS focused exclusively on the ∆BW traits. Four genome-wide significant QTLs and one suggestive QTL were detected for body-weight variations.

To the best of our knowledge, this is the first study describing QTL associated with body-weight variations measured during the adult laying period. In contrast to studies of juvenile growth, the ∆BW traits capture weight changes after sexual maturity, when structural growth is largely completed and body-weight variation is more likely to reflect changes in body composition. Accordingly, the QTLs detected in this study are more plausibly related to genetic mechanisms influencing adult metabolic dynamics and fat deposition rather than general growth processes as supported by the high genetic correlation observed between AFW and ∆BW traits. Notably, the QTL identified on chromosome 11 for ∆BW3070 overlaps with a genomic region previously reported in the AnimalQTLdb (Hu et al., 2022), as being associated with AFW measured in a broiler × layer cross at 9 weeks of age (Ikeobi et al., 2002), further supporting the biological relevance of this locus for adiposity-related traits. This cross-study positional overlap supports further investigation of the region but does not show that the same causal variant or biological mechanism is involved.

None of the QTLs detected for ∆BW3070 or ∆BW3090 overlapped the AFW or BFT QTLs previously detected in the same population (Bédère et al., 2026; Hubert et al., 2026). This absence of overlap does not contradict the high genetic correlations because a genetic correlation summarizes genome-wide additive covariance and may arise from many loci whose individual effects do not exceed GWAS thresholds. Detection was also asymmetric: 9,657 ∆BW3070 records were available for genetic-parameter estimation and 4,327 genotyped hens for its GWAS, compared with 1,113 AFW records. Differences in statistical power, effect sizes, marker LD, and thresholding can therefore prevent shared loci from being detected for both traits. Conversely, a high genetic correlation does not establish that any particular QTL is shared.

Collectively, these findings expand the catalog of loci associated with adult body-weight variation, potentially involved in lipid-related traits in aging laying hens.

GWAS loci were initially defined by fixed ±500 kb windows around lead SNPs, these windows are not causal intervals and can contain many positional genes. LD-guided refinement reduced three of the five intervals by at least 40 %, facilitating candidate prioritization without identifying causal genes or variants. This is consistent with the extent of linkage disequilibrium typically observed in laying hens, where LD blocks span on average around 200 to 300 kb (Hérault et al., 2018). The chromosome 3 QTL for ∆BW3070 was reduced to four lncRNAs and none of which was expressed in liver. Whereas these lncRNAs may play regulatory roles, their functional characterization remains limited, thereby hindering the prioritization of candidate genes (Jehl et al., 2020).

LD-guided refinement was complemented by eQTL mapping to improve candidate-gene prioritization. Similar integrative approaches have been applied in humans to body mass index, rheumatoid arthritis, schizophrenia and low-density lipoprotein traits (Giambartolomei et al., 2014; Zhu et al., 2016), and in mice to breast-cancer risk (Beesley et al., 2020).

### Candidate gene prioritization by eQTL integration and functional annotation

Following LD-based refinement of QTL regions, liver eQTL analyses were conducted to further prioritize the candidate genes underlying GWAS signals. As suggested by the strong genetic correlations observed between ∆BW traits and AFW, body weight variation may represent an indirect genetic indicator of AFW. Under this assumption, genomic regions associated with ∆BW are expected to explain a part of adiposity genetic determinants. It was then assumed that causal variants contributing to lipid-related trait variation were regulatory variants of gene expression in the liver, the central organ of lipid synthesis, mobilization, and export in laying hens. The integration of QTL mapping with hepatic eQTL analyses, therefore, enabled the prioritization of putative causal genes whose expression may be influenced by variants associated with adiposity-related phenotypes. The analysis prioritizes statistical candidates only within this tissue and physiological context. Such an approach requires substantial sample sizes to ensure adequate statistical power and relies on a biologically supported hypothesis regarding the tissue in which the causal regulatory mechanisms are most likely to operate. Compatible QTL-eQTL signals were identified for two of the five ∆BW QTLs.

Within the chromosome 2 QTL for ∆BW3070, *CASD1* and *PDK4* were statistically prioritized by the QTL-eQTL analysis, with PP.H4 values of 0.95 and 0.83, respectively. *PDK4* inhibits the pyruvate dehydrogenase complex and thereby contributes to the regulation of glucose oxidation and fatty-acid utilization (Holness and Sugden, 2003; Wu et al., 1999). Variation in its hepatic regulation could therefore plausibly influence substrate use and adult body-weight dynamics, although this mechanism was not tested here. *CASD1* encodes a sialate O-acetyltransferase (Arming et al., 2011), but no direct evidence currently links *CASD1* to adiposity regulation in poultry. Nevertheless, the established metabolic role of *PDK4* does not by itself justify prioritizing it over *CASD1*. The absence of a currently known relationship between *CASD1* and these traits does not exclude an uncharacterized biological function. *CASD1* and *PDK4* should therefore be regarded as two statistically prioritized candidates supported by different types of evidence: stronger colocalization support for *CASD1* and greater prior functional plausibility for *PDK4*.

Within the chromosome 11 QTL for ∆BW3070, *CMIP* and *PNAT10* were statistically prioritized, with stronger colocalization support for *CMIP* (PP.H4 = 0.88) than for *PNAT10* (PP.H4 = 0.64). *PNAT10* participates in RNA acetylation (Arango et al., 2018), whereas *CMIP* has been implicated in intracellular signaling in other biological contexts (Zhang et al., 2010, 2017). However, neither gene has an established role in adiposity or adult body-weight regulation in poultry. The support for *PNAT10* was comparatively limited and should therefore be regarded as a more hypothetical candidate. However, these two genes require independent molecular and functional evidence.

Overall, *CASD1* and *PDK4* represent the principal candidates within the chromosome 2 QTL, whereas *CMIP* is a strong candidate within the chromosome 11 QTL and *PNAT10* remains more hypothetical. These results prioritize candidates for further investigation but do not establish that any of these genes is causal.

For the remaining QTLs associated with ∆BW3070 and ∆BW3090, no liver eQTL showed a consistent colocalization pattern with the GWAS signals. This absence may reflect limited eQTL power (459 hens) or regulatory effects specific to another tissue, age, or metabolic context, despite the expression of many protein-coding genes in multiple tissues (Degalez et al., 2024; Derrien et al., 2012) and the existence of cross-tissue eQTLs (GTEx Consortium, 2017). Alternatively, these QTLs may influence adult body-weight variation through processes unrelated to lipid deposition, reducing the likelihood of detecting colocalizing liver eQTLs involved in lipid metabolism. Resolving these loci will require additional functional genomic data from relevant tissues, including chromatin accessibility and promoter-enhancer interaction maps. Other mechanisms not assessed here may also be involved, including protein-altering coding variants, alternative splicing (**sQTLs**), transcript usage or 3′UTR-related regulation (**3′ untranslated region QTLs**), and DNA methylation (**meQTLs**), which may be particularly relevant to aging. Although coding variants directly affecting protein structure generally contribute less frequently to complex traits, they cannot be excluded. Overall, integrating genomic associations with gene expression remains valuable for prioritizing candidate genes and guiding functional validation of ∆BW and AFW QTLs.

### Study limitations and future validation

Several limitations constrain interpretation and generalization. All analyses were conducted in one commercial Rhode Island Red line, without cross-validation of ∆BW as a predictor of AFW or external population validation. Sample sizes were markedly unequal: 9,657 hens had ∆BW3070 records, 1,113 had AFW records, and 459 had liver RNA-seq data. The smaller AFW and transcriptomic datasets reduce precision and power relative to ∆BW3070, complicating comparisons of QTL detection and limiting eQTL discovery. The eQTL analysis was restricted to liver at 90 weeks and could not capture regulatory effects specific to adipose tissue, other organs, or earlier ages. In addition, ∆BW is environmentally influenced and combines changes in multiple body compartments. These limitations mean that the results establish genetic relationships and prioritize hypotheses, but do not demonstrate prediction accuracy, causality, or readiness for breeding implementation.

## Conclusion

In this Rhode Island Red population, ∆BW3070 and ∆BW3090 were strongly genetically correlated with AFW, supporting their consideration as potential non-invasive indirect indicators. Their relationships with LR were evaluated and differed depending on the ages used to calculate ∆BW. In contrast, BFT did not follow the same pattern as AFW and showed weaker genetic correlations with ∆BW traits, indicating that ∆BW traits are more specifically related to abdominal fat reserves than to subcutaneous fat deposition. Nevertheless, independent population validation and direct evaluation of predictive ability are required before implementation in breeding programs. GWAS and liver eQTL integration identified associated regions and statistically prioritized *CMIP, CASD1* and *PDK4*, the latter having functional support. *PNAT10* was retained as a more hypothetical candidate, although these analyses did not establish causality.

## CRediT authorship contribution statement

**Alexandre Hubert:** Conceptualization, Methodology, Investigation, Formal analysis, Data curation, Interpretation of results, Writing – original draft. **Nicolas Bedere:** Methodology, Data curation, Interpretation of results. **Fabien Degalez:** Methodology, Formal analysis, Data curation, Interpretation of results. **Laetitia Lagoutte:** Data curation, Supervision, Investigation, Interpretation of results **Coralie Allain, Lorry Becot, Quentin Berger, Thierry Burlot, Alexandre Ferry, Bénédicte Lebez:** Data curation, Investigation, Interpretation of results **Mathieu Emily:** Methodology, Interpretation of results. Data curation, Investigation. **Marie-Pierre Sanchez:** Methodology. **Thierry Tribout:** Methodology, Interpretation of results. **Frédérique Pitel:** Conceptualization, Methodology, Interpretation of results. **Tatiana Zerjal:** Conceptualization, Methodology, Interpretation of results, Writing – original draft. **Sophie Allais:** Conceptualization, Methodology, Data curation, Interpretation of results, Writing – original draft. **Sandrine Lagarrigue:** Conceptualization, Methodology, Investigation, Data curation, Formal analysis, Interpretation of results, Writing – original draft.

All authors reviewed and approved the final version of the manuscript.

## Ethics approval and consent to participate

All data were collected as part of the Novogen (Plédran, France) breeding program. Data collected from live animals were genotypes and phenotypes for egg number, egg weight, body weight, liver weight and abdominal fat weight. Phenotypes for abdominal fat weight and liver weight were collected post mortem after animals were euthanized in accordance with national animal production regulations and procedures approved by the French Veterinary Services.

## Ethics declaration

### Animal subject

This study was conducted in accordance with the ARRIVE (Animal Research: Reporting of In Vivo Experiments) guidelines. This study was approved by the French Veterinary Services.

## Disclosures

The authors declare the following financial interests/personal relationships which may be considered as potential competing interests: Thierry BURLOT reports a relationship with Novogen that includes: employment. Lorry BECOT reports a relationship with Novogen that includes: employment. Alexandre FERRY reports a relationship with Novogen that includes: employment. If there are other authors, they declare that they have no known competing financial interests or personal relationships that could have appeared to influence the work reported in this paper. None of the commercial activities and interests of Novogen influenced the research in any matter.

## Data Availability

The data that support the findings of this study are available from Novogen but restrictions apply to the availability of these data, which were used under license for the current study, and thus are not publicly available. Data are however available from the authors upon reasonable request and with permission of Novogen (thierry.burlot@novogen-layers.com). Raw data of RNA-seq are publicly available on the ENA and FAANG portal under accession numbers PRJEB63475.
